# Indents

**Published:** 1905-10-15

**Authors:** 


### INDENTS.

---

**☞ Brief Articles of Technical or Scientific Value will receive notice and comment. Contributions invited.**

---

**Nose Bleed.** Grasp the nose between the thumb and forefinger, and press backwards against the alveolar border of the maxilla and downward against the teeth. This compresses the lateralis nasi and septal arteries. Satisfactory results also follow the use of tannin and acetanilid.—*Laryngoscope.*

**Pyorrhea Alveolaris.** If every particle of irritant and foreign matter was removed from about the teeth more than 70 percent of the cases of so-called pyorrhea would get well of themselves with no other therapeutic treatment than an alkaline mouth-wash. Successful practice means careful and thorough manipulation, and no amount of therapeutic treatment can supplement it or take its place.—F. G. Worthly, *Western.*

**Agreeable Toothache Cure.** Robert Clarke, the artist, tells this story: "One day, while out walking with a friend of his, this friend complained of a tooth-ache and asked Mr. Clarke what he could advise him to buy, as they were in front of a drug store. 'Why,' said Clarke, 'the last time I had a tooth-ache I went home and my wife kissed it away for me.' After a moment's pause his friend said: 'Is your wife home now?' "—*Digest.*

**Recommending Nostrums.** A few years ago a fellow came into my office, introduced himself and asked for time to demonstrate a method. I had always prided myself on being proof against these fellows, but this one was too much for me, and after an exausting siege, in which he employed everything from heavy artillery to a cannon fire-cracker, I surrendered and paid him the money. I also think he gave me a demonstration of his method, but on this point I am not quite sure. After we were all through he asked me as a special favor to give him a letter of recommendation, and just to show that I was no easy mark I gave it to him. Just what he did with that letter I do not know, but I am satisfied he didn't burn it, for in a short time I began to hear from my friends over the state, usually in no moderate terms. One asked me if I got a percentage; another wanted to know where I put the time I saved by using this method, and one sober-minded friend asked if I had ever been in the lightning-rod business. It kept me busy for a long time squaring myself, and I am afraid my reputation for good judgment has been unalterably tarnished, but the experience was worth the money—to me.—F. R. Henshaw, in *Digest*.

**Value of Association.** I believe that one of the greatest helps a young man can receive is by forming acquaintances and by association with the best men of his profession. A good many of the young men of this society can look back and remember how they appreciated the kind words spoken to them by the older members when they first began to attend these meetings. I personally remember two incidents especially that took place at these meetings several years ago which made indelible impressions on my mind. One occurred at the first meeting I attended, when the late Dr. S. B. Brown extended his greetings, asked how I was getting along and if I was meeting with any obstacles in my practice in which he could be of any service to me, assuring me that if at any time I should like his advice he would be glad to give same. Not long afterward I had occasion to write him, and I wish you could have read his reply, but you who knew him realize that it was full of instruction and encouragement. The other incident occurred at a meeting which Dr. Black, of Chicago, attended, and when I went home I could not help but think that had I not gained anything else at that meeting but the half hour's conversation with the Doctor, I would have been well repaid for attending. Think of the contrast between meeting such men and those who never pronounce their own names without involuntarily taking off their hats, so profound is their self-admiration.—C. G. Keehn, in *Digest.*

**The National Meetings, 1905.** When the writer first essayed to comment upon the recent meetings at Buffalo, there came to mind many things to criticise, and so he criticised. There was much that he thought needed mending—and so he suggested how to amend. He was caustic regarding the confusion—upon the new sectional arrangement—upon a thousand and one things that seemed "gone awry"—and then the manuscript went to the waste basket because he came across, in a daily newspaper which is republishing the writings of Seneca, the following: "*We are ready enough to limit ourselves, but loath to put bounds and restraint upon ourselves.*" Perhaps, the writer thought, each individual present at that meeting criticised the conduct of the meetings from his own certain standpoint, criticised the arrangements, criticised rulings of the officers, criticised individuals, criticised individual efforts, criticised the essays, the clinics and what not, but did not go quite far enough, and *criticise himself.* This laying of bounds for others and not "putting restraint upon himself" may have been the cause of every untoward thing seen at the meetings. If any one active individual had seen fit to do work possible for him toward the completeness of each one of these meetings, perchance the things seen that were out "of tune" might have only shown harmony. The critic is a supreme person—in his own mind. "Many a man whirls in to reform the world, only to discover at last that he didn't know how to reform himself," says the Atlanta *Constitution,* and we think it especially applicable to some critics of the late meetings. One editorial writer, in commenting upon the adoption of the report of the Committee on Schools of the Faculties' Association, grows pessimistic at the action taken. At the meeting his objection had only one adherent, and every other member agreed that the recommendations of the committee made a great step towards the solution of the chief difficulties and wranglings which have in past years hindered dental progress. We suggest that possibly the Atlanta *Constitution's* man referred to those who are given to just such caprices. Another critic, commenting upon the papers presented at the National, advises a "properly constituted board of censors" to cut from the program efforts not up to standard. This critic maybe forgets that we live under a form of government which discourages autocratic powers and whose people look askance at anything that will abridge their "God-given rights;" and fails to appreciate that possibly the objectionable existing custom, if dominated by a "board of censors," would lead to "*confusion worse confounded.*" But we are digging up matter consigned to the waste-basket—comments that no doubt others will think should, too, require a censorship.

One thing, however, we can say respecting the Buffalo meetings, and that is that the visitors received never-to-be-forgotten courtesy, attention and entertainment from the good Buffalo people.—*Western Dental Journal.*

**"What's in a Name?"** If we judge from the papers and discussions heard at associations and read in dental journals, there must be a great importance in names; as to the real and vital situation, there may be some doubts. At the recent National meeting in Buffalo, a prominent member, in deliberate stilted phrase, and at much length, discussed the well-worn argument respecting the difference in the meaning of the words "occlusion" and "articulation," as if the permanency and life of dentistry depended upon a distinct understanding of that difference. The weary listener who had, perhaps, traveled thousands of miles for information, must have given a sigh of relief when he concluded. When we calculate the valuable time occupied by speakers at dental meetings, and the cords of space in journals, elucidating the subject of a proper term for what was long known as "Rigg's Disease,"we may be excused if we doubt the horse sense of many in the dental profession. A reasonable and self-explanatory nomanclature is what all science demands; but when every cross-road doctor, in order to show his patent leathers or white vest and get his name in the proceedings, meanders over the fallow field of disputed terms, he generally brings a feeling of intense lassitude to his audience.

In the same category may be classed the man imbued with the too prevalent idea that the advancement of our profession depends upon the number of high-sounding terms we use. This is a curious squint, but it is a fact. While sitting in the corridore of the Iroquois, at Buffalo, at our last national meeting, two dentists—no beg pardon, two *stomatologists*—were overheard in conversation, and they, while not agreeing on many points, did thoroughly agree that it was a great and up-building thing that we now "hear nothing more of mechanical dentistry;" "that 'prosthetic' and 'prosthesis' were terms showing better education!" By all means adopt the new , if better; but, for the harmony found in proper appreciation, let us not argue that values and good morals are dependent upon how named.

Long have been the arguments favoring the adoption of the word "stomatology" to indicate the field of the dental practitioner; and the self-chosen elect in many bailiwicks have formed "stomatological clubs" galore, and pride themselves upon being in the advance by virtue of the name and argue that by this adoption they are less of the mechanic and more artistic and professional. They forget that mechanics *hold highest place* in our new world, and that taking new names, or false names, "*is a kind of visard whereby men disguise themselves.*"

Although from time immemorial the name "pedagogue" has been defined as a teacher, "*especially one who teaches*

*in a conceited, pretentious way*" (Standard Dictionary), yet the dental teachers of the United States changed the name of the "National Dental Technic Association" to the "*Institute of Dental Pedagogics,*" because it would be a more "dignified title." The word "technic" savored too much of mechanics to suit those who as Hogg has said, were teachers "too much in the pedagogue class." Can any sane man deny that "The National Institute of Dental Teachers" would be preferable to "The Institute of Dental Pedagogues?" or can there be comparison between the title "New York Institute of Stomatology" and, say, the "New York Institute of Dental Science?" What is the more dignified, which is the more cultured title? "What's in a name?" Much in a good and proper one.—J. G. Patterson, in *Western Journal.*

